# Intratesticular Abscess in a Solitary Testicle: The Case for Testicle Sparing Management

**DOI:** 10.1155/2013/184064

**Published:** 2013-10-03

**Authors:** Uwais B. Zaid, Herman S. Bagga, Adam C. Reese, Benjamin N. Breyer

**Affiliations:** Department of Urology, University of California San Francisco, San Francisco, CA 94143, USA

## Abstract

We present the case of a 24-year-old immunocompromised man with an intratesticular abscess. The patient presented with one week of left scrotal pain and swelling. Workup included scrotal ultrasonography which revealed a large fluid collection within the tunica albuginea of the left testis. Surgical exploration of the left testis evacuated a significant amount of purulent fluid. The residual viable testicular parenchyma was salvaged. Intraoperative cultures grew *Morganella morganii.* Follow-up ultrasonography showed resolution of the testicular fluid collection, and the patient was discharged home with oral antibiotics. Intratesticular abscesses are a rare clinical entity which often result in orchiectomy.

## 1. Case Presentation

A 24-year-old male presented to the emergency department with one week of left testicular pain and hemiscrotal swelling. He denied fevers, chills, nausea, vomiting, dysuria, and hematuria. Of note, nine months prior to presentation, he was found to have a right scrotal abscess that was consuming the right testicle, requiring incision and drainage and right orchiectomy. The patient's past medical history is significant for HIV (CD4 count 348, viral load >200,000, not compliant with his ARV medications), polysubstance abuse, syphilis, and herpes zoster.

**Figure 1:**
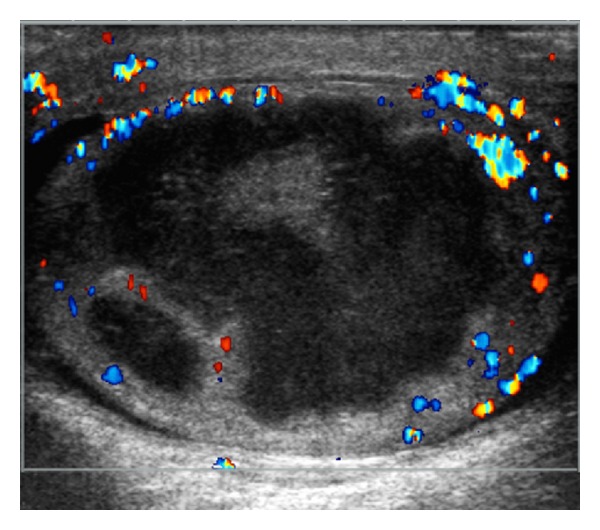
Scrotal ultrasound at presentation.

On presentation, he was afebrile with normal vital signs. Physical examination revealed a warm, erythematous, swollen left hemiscrotum that was tender to palpation. DRE revealed a nontender, normal-sized prostate. A white cell count was 9. We did obtain a testosterone level which was 52 ng/dL. Urine and blood cultures were negative. A scrotal US (Figure 1) revealed a markedly heterogenous left testicle measuring 5 × 3.5 × 3.7 cm with a large central hypoechoic region with a rim of markedly hypervascular testicular parenchyma. The epididymal head was noted to be heterogeneous with markedly increased vascularity. This was consistent with epididymo-orchitis with an intratesticular abscess.

We elected to admit the patient and started him on IV vancomycin and ertapenem. On hospital day three, the patient remained afebrile and his white cell count remained within normal limits with an unchanged clinical examination. We thus obtained a repeat scrotal ultrasound, which remained largely unchanged.

**Figure 2:**
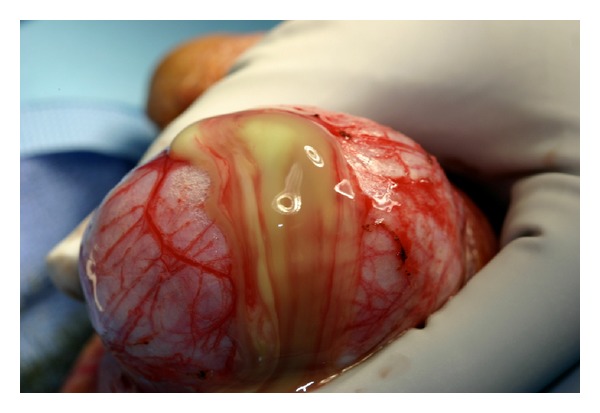
Intraoperative photo demonstrating extravasation of purulent fluid from left testicle.

**Figure 3:**
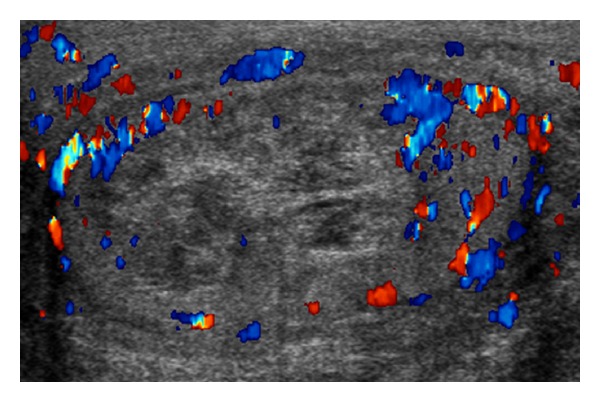
Postoperative scrotal ultrasound.

We thus elected to take the patient to the operating room on hospital day four, and he underwent a left hemiscrotal exploration. The tunica vaginalis was noted to be under significant pressure, and upon opening the tunica albuginea we noted extravasation of a significant amount of purulent, foul-smelling fluid (Figure 2), which was sent for AFB and aerobic and anaerobic cultures. We did note significant viable parenchyma of the left testicle. After copious irrigation, a tiny little sucker (TLS) drain was placed into the testicular parenchyma (Figure 3).

The patient's drain was discontinued on postoperative day two after minimal output. On postoperative day four, testosterone level was 63 ng/dL. A repeat ultrasound at this time revealed no abscess or fluid collection. The AFB culture was negative; however, the bacterial culture grew *Morganella morganii*. The patient was discharged home on postoperative day four with a four-week oral antibiotic regimen.

He was unfortunately lost to followup, and no further imaging or testosterone levels have been obtained since discharge.

## 2. Discussion

Intratesticular abscesses are a rare clinical entity. These are usually associated with advanced or untreated epididymo-orchitis, most commonly caused by *E. coli* that may spread hematogenously or by reflux of urine. Up to 5.5% of untreated epididymo-orchitis will progress to involve the testicular parenchyma [1]. Patients who are immunocompromised or diabetic are at increased risk [1, 2]. Over 50% of cases result in orchiectomy [1, 2]. Scrotal ultrasonography is the ideal imaging modality. [3] Given this patient's prior history of orchiectomy we elected to proceed with a testicular sparing approach. Interestingly, microbiology showed *Morganella morganii*.
